# Sexual counseling for people with acute coronary syndrome: educational video development

**DOI:** 10.1590/0034-7167-2023-0416

**Published:** 2024-07-29

**Authors:** Laura Rizardi dos Santos Andrade, Lanay Dourado dos Anjos, Ana Paula Freitas Aguiar, Edvone Alves de Lima, Erika de Sá Vieira Abuchaim, Juliana de Lima Lopes, Camila Tákao Lopes, Vinicius Batista Santos

**Affiliations:** I Universidade Federal de São Paulo. São Paulo, São Paulo, Brazil; II Hospital Sírio-Libanês. São Paulo, São Paulo, Brazil; III Hospital Universitário São Paulo, Unidade de Cardiologia. São Paulo, São Paulo, Brazil; IV Hospital Israelita Albert Einstein. São Paulo, São Paulo, Brazil

**Keywords:** Sex Education, Audiovisual Aids, Cardiovascular Disease, Health Education, Validation Study, Educación Sexual, Recursos Audiovisuales, Enfermedades Cardiovasculares, Educación en Salud, Estudio de Validación

## Abstract

**Objective:**

to assess validity evidence of an educational video on safe sexual activity after acute coronary syndrome.

**Method:**

study in three phases: video development; content validity analysis by 11 experts; and analysis of validity based on response processes by seven people with coronary disease. The content validity ratio (CVR) was calculated with critical values for the second phase of 0.63 and for the third of 1.0.

**Results:**

the video addressed the importance of resuming sexual activity and positions that consume less energy, clinical warning signs, the importance of adhering to treatment and a welcoming environment for sexual practice. A CVR above the critical value was obtained with a total of 4 minutes and 41 seconds.

**Conclusion:**

the educational video brings together adequate content validity evidence and can be used as a tool for patients after acute coronary syndrome.

## RESUMEN

**Objetivo:**

evaluar la evidencia de validez de un video educativo sobre actividad sexual segura después del síndrome coronario agudo.

**Método:**

estudio en tres fases: elaboración de un vídeo; análisis de validez de contenido por 11 expertos; y análisis de validez basado en procesos de respuesta de siete personas con enfermedad coronaria. Se calculó el índice de validez de contenido (CVR) con valores críticos para la segunda fase de 0,63 y para la tercera de 1,0.

**Resultados:**

el video abordó la importancia de retomar la actividad sexual y posiciones que consuman menos energía, signos de alerta clínica, la importancia de adherirse al tratamiento y un ambiente acogedor para la práctica sexual. Se obtuvo un CVR por encima del valor crítico con un total de 4 minutos y 41 segundos.

**Conclusión:**

el video educativo reúne evidencia adecuada de validez de contenido y puede ser utilizado como herramienta para pacientes post síndrome coronario agudo.

## INTRODUCTION

Cardiovascular diseases (CVD) can compromise the life of a person and their family, both physiologically and psychosocially. People who have suffered acute coronary syndrome (ACS) report changes in quality of life and increased dependence, in addition to social isolation and evolution to states of frailty as the disease progresses. One of the negative repercussions of CVD that directly impacts psychosocial aspects is sexual dysfunction^(1-3)^.

sexual function response was initially described by Willian Master and Virginia Johnson in four phases, namely excitement, plateau, orgasm and resolution. In the 1970s, Helen Kaplan understood that, before excitement, there is desire, therefore, she eliminated the plateau phase, proposing a human sexual response three-phase model, covered by the desire, excitement and orgasm phases, which can be applied in both men and women. Based on previously described human sexual response models, the International Classification of Diseases and the Diagnostic and Statistical Manual of Mental Disorders described the sexual function response cycle linearly in four phases: desire; excitement; orgasm; and resolution. Influenced by biological, psychological, social, historical and cultural factors, this four-phase linear model is most characteristic of male sexual response^(4-5)^.

In 2005, Basson described a circular model of overlapping phases that characterizes female sexual response. In this model, starting from sexual neutrality, but motivated and receptive to sexual stimulation, women trigger subjective excitement. The experience of subjective excitement leads to sexual desire and, with the gradual increase in excitement and desire intensity, sexual pleasure occurs, with or without orgasm, leading to physical and emotional satisfaction^(5-6)^.

Several types of sexual dysfunctions affect the general population due to a lack of sexual desire, excitement and/or difficulties in reaching orgasm. However, there are particularities related to the biological differences presented by individuals, i.e., people with a vagina or a penis may have a higher prevalence of sexual dysfunction in different phases of sexual response^(7)^.

The literature demonstrates, in the general population, a prevalence of 33.4% of sexual problems in people with a penis^(8-9)^ and 45.7% in people with a vagina^(10)^. In people with a penis, the sexual dysfunctions that cause the greatest distress are erectile dysfunction and premature ejaculation^(8-9,11)^. In people with a vagina, the most commonly reported sexual dysfunctions are related to hypoactive sexual desire and orgasmic dysfunction, with the main complaint being pain^(10-11)^. However, in people with CVD, these numbers tend to rise, reaching up to 90% in women and 79% in men^(11)^.

It is known that women can experience sexual disorders in the face of excitement, a process made up of a series of behavioral, hormonal and morphological mechanisms and characteristics. There is evidence that CVD and metabolic diseases are associated with changes in vaginal tissue structure and function, impacting these women’s sexual performance^(11)^.

Erectile dysfunction in men has a strong relationship as a predictor of CVD and subclinical conditions in asymptomatic men. Thus, it represents a potential opportunity for CVD screening in men with a focus on early detection and prevention of harmful events, therefore reducing morbidity and mortality. Furthermore, some antihypertensive medications commonly used in people with CVD can negatively interfere with the erection process itself^(12-15)^.

As sexual health is a topic to be considered in the general population and, in particular, in those diagnosed with a CVD, health education plays a fundamental role in allowing effective guidance so that this practice can be carried out satisfactorily and with safety. Health education is a strategy for promoting patient care with the aim of preventing and mitigating complications caused by a given disease, considering patients as active and essential agents for effective treatment, in order to provide information and guidance relevant to the case, aiming to promote autonomy and a sense of responsibility for one’s own clinical condition and the decisions to be made regarding treatment^(16-17)^.

Studies reveal that healthcare professionals demonstrate difficulty in addressing aspects of sexuality in patients with cardiovascular dysfunction, especially among those post-hospitalization for ACS^(18-20)^. Health education can be carried out in different practice scenarios for professionals, such as home visits, groups, workshops, videos and even with the distribution of educational materials, such as educational booklets and even blogs, websites and social networks. Studies on health education opted for the video format for several reasons, such as easy distribution and access, visual stimuli that allowed greater patient focus and obtained positive results on their involvement within treatment regimen^(21-22)^.

In the cardiology field, research produced an educational video aimed at patients who would undergo cardiac catheterization. Among the main results, patients had greater knowledge about factors related to the procedure and current health status and had lower levels of anxiety compared to control group patients. This intervention model presented several advantages for patients’ health literacy, as it could be viewed at the desired speed and number of times, facilitating knowledge transmission^(23)^.

In a survey carried out by the authors, no studies were identified that developed and validated an educational video to promote safe sexual activity for patients who were hospitalized for ACS, and it is believed that this educational video can be implemented as an educational tool for this population.

## OBJECTIVE

To develop and assess validity evidence for an educational video on safe sexual activity after ACS.

## METHODS

### Ethical aspects

This project was approved by the university’s Research Ethics Committee (REC), in accordance with Resolution 466/12 of the Brazilian National Health Council/Ministry of Health. All participants were informed, through the Informed Consent Form (ICF), regarding the research objective and voluntary aspect as well as data collection procedures. Anonymity and confidentiality were guaranteed to participants.

### Study design and place

This is a methodological study of development and analysis of validity evidence for an educational video on safe sexual practice after ACS. The study was carried out in three phases: 1) video development; 2) analysis of content validity evidence according to expert opinion; and 3) analysis of validity evidence based on response processes by a group of patients hospitalized for ACS in a large university hospital in the city of São Paulo, containing ten cardiology inpatient beds and six intensive care beds.

### Sample and inclusion criteria

In the second phase, healthcare professionals who carried out research in relation to sexual health promotion or with experience in the health education process for patients with CVD participated.

In the third phase, the video previously assessed by experts was presented and assessed by lay people, literate, over 18 years old, hospitalized in a large hospital in the city of São Paulo due to ACS and who were not hemodynamically unstable, with recurrence of chest pain or using vasoactive drugs.

The minimum sample size in the second and third phase was based on Lawshe’s recommendations to obtain a critical content validity ratio (CVR) for adequate agreement among experts, with a minimum of five experts^(24)^.

### Study protocol

When seeking to conceptualize the problem, the first phase was based on a narrative literature review that aimed to map and identify the main guidelines for promoting the sexual life of people after ACS.

Study search and selection were guided by the SPIDER technique: Sample: adult patients over 18 years old and with CVD; Phenomenon of Interest: guidelines for promoting sexual life; Design: intervention studies (clinical trials, randomized or non-randomized, pragmatic clinical trials and quasi-experimental studies); Evaluation: promotion of sexual activity; Research type: observational studies, reviews and guidelines^(25)^.

The descriptors “Sex Education” and “Cardiovascular Disease”, in English, and “*Educação Sexual*” and “*Doença Cardiovascular*”, in Portuguese, were used in the PubMed and Virtual Health Library data sources. Studies published between 2016 and 2022 were filtered.

After assessing studies retrieved through critical reading of titles and abstracts, they were read in full by a researcher, and the following data was extracted: title; authors; year of publication; objective; study design; and non-pharmacological guidelines for safe sexual practice.

After identifying non-pharmacological measures for safe sexual practice, a video script was created by two of the researchers in this study with experience in cardiology and sexuality studies. The storyboard-type script developed contained two fictional characters, one of which was a patient and the other a nurse, and the educational video was subsequently developed through the RenderForest^®^ Premium website. The video addressed the importance of sexual health in quality of life and health and the importance of safe sexual activity, highlighting the main non-pharmacological guidelines for maintaining a healthy and functional sexual life in the face of the limitations imposed by the disease.

In the second phase, the educational video was submitted for content assessment by experts. Potential participants were selected by the *Lattes* Platform and, with the authors’ knowledge, an invitation to participate in the study was sent by email and an ICF was sent via a link via Google Forms^®^. Experts assessed the video with regard to clarity, theoretical relevance and practical relevance using a Likert scale (1 = completely disagree, 2 = partially agree and 3 = completely agree).

Theoretical relevance was defined as “the ability of the item to be consistent with the defined attribute and with other expressions that relate to the same attribute”, clarity, as “the ability of the item to be intelligible, with short sentences, simple and unambiguous expressions”, and practical relevance, as “elaboration in order to assess the concept of interest in a given population”^(26)^.

In the third phase, potential participants hospitalized in the cardiology inpatient unit were approached by the researcher during their hospitalization in the cardiology unit, and the study objectives were explained. To those who agreed to participate in the research, the ICF was applied. Subsequently, the video was presented to patients using a tablet and a headset, and they were asked to assess it in relation to scene and information clarity, video usefulness, video running time and understanding using a Likert scale (1 = completely disagree, 2 = partially agree and 3 = totally agree). To complement assessment, patients were asked their opinion about the video and the topic covered, and their responses were transcribed verbatim.

### Analysis of results, and statistics

In the second phase, for each item in the video assessed by experts, CVR was calculated as follows:


$$CVR=\frac{ne-(N/2)}{N/2}$$

Hence, *ne* represents the number of experts who scored 3 and *N* relates to the number of experts^(24)^. Based on feedback from 11 experts, the acceptable critical CVR was 0.63 (p. 0.03)^(24)^.

In the third phase, to analyze validity evidence based on response processes, CVR was calculated, as previously described, and, according to the assessment of the seven participants, the critical CVR was 1.0 (p. 0.008)^(24)^.

## RESULTS

Initially, 385 articles were identified in the narrative literature review, 62 of which were assessed for complete reading of articles. After critically reading the studies, four references^(27-30)^ were used with guidelines for resuming safe sexual activity after ACS, all of whom cited the environment, medication adherence and positions that required less energy expenditure as guidelines for sexual practice.

The main guidelines from articles are related to a safe environment, the importance of medication adherence, physical activity, healthy eating and recognizing warning signs, being rested for sexual activity, opting for positions that require less energy expenditure and facilitate breathing, gradual resumption of sexual practice, in addition to guidance regarding the need for communication regarding this topic with healthcare professionals^(27-30)^. Chart 1 provides other information about the articles included in this study.

**Chart 1:** Synthesis of guidelines related to sexual practice. São Paulo, São Paulo, Brazil,2022

Ref	Study objective and design	Research findings and types of counseling
27	Provide essential information for healthcare professionals to initiate practical and applicable sexual counseling for men and women with CVD. Review study	Medication: establish a good relationship with patient, guide medication adherence, assess the root of the dysfunction and possibilities for changing or adjusting the dose; Environment: comfortable and familiar to minimize cardiac stress; Warning signs: advise reporting any signs and symptoms; Physical training: exercising regularly to reduce the risk of cardiac events; Intensity and energy consumption: dependent on physical capacity and being well rested before sexual activity; Positions: must allow comfort and facilitate breathing.
28	Provide practical, evidence-based approaches to enable healthcare professionals to discuss sexual counseling, illustrated by various case scenarios. Review study	Medications: advise reporting any side effects and not stop after one appears; Environment: comfortable and familiar, rest before sexual activities, avoid heavy meals and excessive alcohol consumption before sexual intercourse; Position that provides better comfort; Warning signs: chest pain, extreme shortness of breath, elevated or irregular heart rate, fatigue the day after sexual activity, dizziness or insomnia; Activity: regular physical activity as tolerated; Resumption of sexual activity: start with activities that require less intensity/energy expenditure, such as hugs, kisses, caresses.
29	Describe the latest guidelines published by the American Heart Association on sexual activity in patients with heart disease as well as treatment options for sexual dysfunction. Review study	Clinical assessment (low risk of cardiac complications); Decompensated patients should abstain from sexual activity until stabilization; If any type of sexual dysfunction is noted due to medications, they should not be suspended and complaints should be assessed to rule out other sources; Be rested before sexual activity, avoid unfamiliar places and sexual partners to reduce stress; Avoid heavy food and alcohol; Preference for positions that facilitate inspiration.
30	Present a comprehensive literature review describing the sexual problems of women after myocardial infarction. Review study	Lack of knowledge among professionals, low prevalence of guidance on sexual function; Educational programs for sexual therapy: patient education, cognitive restructuring, emotional support, medical guidance, increase frequency and quality of sexual activities after myocardial infarction; Psychological factors (such as anxiety) worsened during physiological changes during sex.

Based on the survey of issues related to sexual orientation after ACS, a storyboard was initially created containing two main fictional characters, one of them being a patient after hospital discharge, called Pedro, and a nurse, Julia, who covered the topics mentioned in Chart 1. Subsequently, the video was created in RenderForest^®^ Premium, containing the lines, images of characters in 3D and narration, totaling 34 screens, lasting 3 minutes and 48 seconds.

After developing the video, an invitation was sent to 19 experts, with feedback from 11 experts, 100% of whom were women with a mean age of 32 years; 90.9% were nurses; 9.1% were nutritionists; 27% had a *stricto sensu* graduate degree; 72% had specialization; 90% had experience in cardiology; and 27% had experience in cardiology and sexuality. In the overall assessment of the video in the first round, a CVR of 0.4 was obtained in relation to the amount of information, 0.8 for running time and usefulness, and 1.0 for practical relevance of video information. In the analysis of each scene, only two screens did not present a critical CVR value in terms of clarity, three in relation to relevance, and four in relation to practical relevance.

The main suggestions in the first round were in relation to better contextualization of the initial scene, writing and spelling, the need for the nurse to introduce herself at the time of appointment, inclusion of a minimum weekly time for carrying out physical activity, welcoming regarding doubts regarding sexual activity, better explanation of sexual positions and clarification of medications and the risk of use without the doctor’s consent and taking action regarding the appearance of ischemic symptoms during and after sexual activity.

After modifying the video, it was sent back to the same group of experts for new assessment, and a CVR 1.0 was obtained in the overall assessment in relation to the amount of information, running time and usefulness. In the analysis of each scene, a CVR greater than 0.80 was obtained in all scenes. The mean CVR achieved in this round was 0.99 for clarity, 0.99 for theoretical relevance and 0.98 for practical relevance. Table 1 shows the video and final CVR for each scene in relation to clarity, theoretical relevance and practical relevance.

**Table 1:** Final version of the video and content validity ratio in relation to the assessed indicators. São Paulo, São Paulo, Brazil, 2023

Content	Cla	The	Pra
Let’s talk a little about Pedro’s concerns about sexual activity after leaving the hospital	1.0	1.0	1.0
**At a gathering of friends**	1.0	1.0	1.0
**Friend:** My friend Pedro, how are you feeling after leaving the hospital?	1.0	1.0	1.0
**Pedro:** I’m fine, but can I have sex again?	1.0	1.0	1.0
**Friend:** I think you should talk to a professional about this at your next appointment and wait to have sex again.	1.0	1.0	1.0
**Pedro:** I think I’ll wait and ask at my next appointment.	1.0	1.0	1.0
**On the day of the appointment**	1.0	1.0	1.0
**Nurse:** Good morning, Pedro! I’m nurse Júlia. Nice seeing you again. How are you?	1.0	1.0	1.0
**Pedro:** I’m doing well. Taking all the medicines the doctor prescribed for me.	1.0	1.0	1.0
**Pedro:** I started walking 30 minutes in the morning every day. I’m feeling great.	1.0	1.0	1.0
**Pedro:** And I’m also eating low-salt foods, avoiding fried foods and eating more fruits and vegetables.	1.0	1.0	1.0
**Pedro:** But there’s something I’d really like to ask and I don’t know how.	1.0	1.0	1.0
**Nurse:** Pedro, remember that I’m here to help you get back to your life safely after your heart attack.	1.0	1.0	1.0
**Pedro:** I’m scared of having sex again. What should I do?	1.0	1.0	1.0
**Nurse:** Look, Pedro, this is a subject that we can talk about calmly and clearly, without judgment or anything like that.	1.0	1.0	0.8
**Nurse:** It is recommended that you wait at least seven days after discharge to resume sexual activities.	1.0	1.0	1.0
**Nurse:** It’s important that you start slowly with touching, kissing and other forms of pleasure that you enjoy that use less energy. This foreplay can increase sexual desire for both you and the person you are with.	1.0	1.0	1.0
**Nurse:** An alternative to spending less energy is to stay in a position where you move less, i.e., prefer to stay underneath.	0.8	0.8	0.8
**Nurse:** It doesn’t matter what sex you are. See in the photos that the colorful person is the one who had the heart attack. So, it is recommended that you stay underneath.	1.0	1.0	1.0
**Nurse:** We also need to know how to recognize, in our own body, what the warning signs are.	1.0	1.0	1.0
**Nurse:** Be aware if you feel very tired, chest pain, and shortness of breath during or after sexual activity.	1.0	1.0	1.0
**Nurse:** If you experience these symptoms and they improve after sexual intercourse, you should see the doctor or nurse who is taking care of you as soon as possible.	1.0	1.0	1.0
**Nurse:** And try to reduce efforts during sexual intercourse until you talk to these professionals.	1.0	1.0	1.0
**Nurse:** But if you don’t improve after rest, go to the emergency room immediately.	1.0	1.0	1.0
**Nurse:** We have other tips that are also important. Write it down, Mr. Pedro:	1.0	1.0	1.0
**Nurse:** To improve your sexual relationship: choose a quiet and calm environment; try to be relaxed and rested beforehand; have a light diet before sexual intercourse; and look for positions that use less energy.	1.0	1.0	1.0
**Nurse:** The doctor will prescribe several important medications for you, and some may have some bad side effects, such as	1.0	1.0	1.0
**Nurse:** Lack of desire to have sexual intercourse, difficulty keeping the penis hard and little lubrication in the vagina.	1.0	1.0	1.0
**Nurse:** But don’t stop following treatment, and if there is a problem, just tell the nurse or doctor who takes care of you.	1.0	1.0	1.0
**Nurse:** For those who need it, lubricants can help with vaginal dryness and also with pleasure itself.	1.0	1.0	1.0
**Nurse:** There are some medicines that can help the penis get hard, but these medicines can be dangerous if used without a doctor’s approval.	1.0	1.0	1.0
**Pedro:** Ah, after this information, I feel calmer, thank you for everything.	1.0	1.0	1.0
**Nurse:** That’s right, Pedro! You are doing great and taking the best care of yourself.	1.0	1.0	1.0
For more information, visit the website: www.educacor.unifesp.br	1.0	1.0	1.0

*Cla: clarity; The: theoretical relevance; Pra: practical relevance.*

After the video was analyzed by experts and presented adequate content validity evidence, it was demonstrated to seven people hospitalized for ACS with a mean age of 61 years, 57% of whom were male patients. Among the indicators assessed in this phase, we obtained a CVR equal to 1.0 for scene clarity, information clarity, video usefulness, running time and understanding. When asked about their opinion of the video and the topic of sexuality, patients reported positive feelings regarding the video and the approach taken.

**Figure 1:**
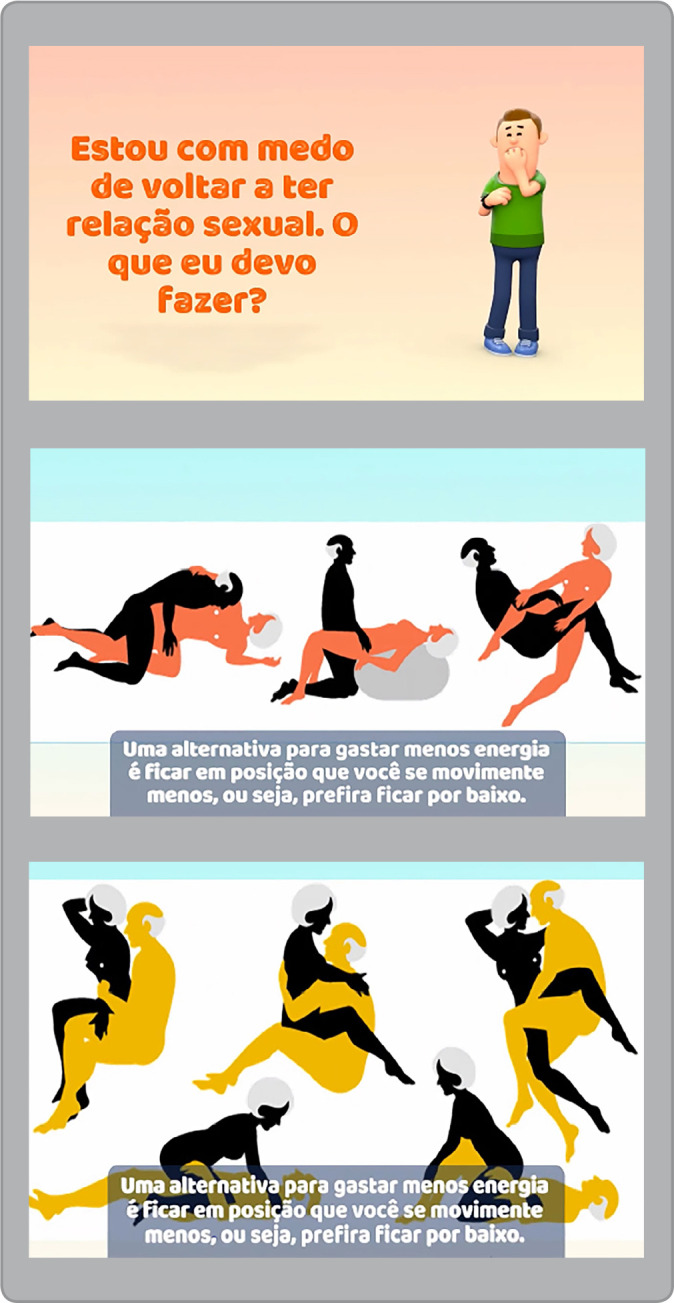
On-screen examples from educational video on safe sexual activity after acute coronary syndrome

In the Figure 1, there are some screens of the educational video developed to guide patients regarding safe sexual activity resumption.

**Figure 2:**
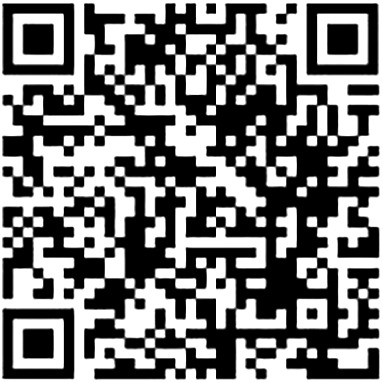
QR code for the educational video on safe sexual activity after acute coronary syndrome

The educational video on safe sexual activity after ACS can be watched using the QR code (FIgure 2).

## DISCUSSION

According to the World Health Organization, sexuality is one of the essential dimensions of human life and involves multiple concepts, such as sex, sexual orientation, gender identity, eroticism, pleasure, intimacy, reproduction and healthy relationships. Its complexity and influence on people’s lives are determined by biological, psychological, social, economic, political, cultural and religious factors, in addition to individual experiences and personal values. Sexuality is constructed from the context in which people live and the interactions they establish with the environment in which they find themselves^(31)^.

Knowing the importance of the human sexual dimension and its impacts on quality of life, it is important that professionals are properly qualified to provide the necessary guidance for each patient’s query. It is possible to identify studies that demonstrated that professionals are not in the habit of discussing sexuality with patients, either because they do not feel comfortable and do not consider sexuality as a priority in care or because they have not actually undergone training to address this issue^(32-33)^.

In a study carried out in Turkey with 170 nurses, it was identified that nurses did not address sexuality in patients with CVD because they did not consider sexuality a priority in patient care, because of a lack of privacy in the inpatient unit, because they considered the subject very personal and due to cultural and religious problems^(18)^. Other studies have also identified several difficulties in sexual counseling in this population, such as a study in Jordan, in which nurses feel they have low confidence when approaching sex, and a survey carried out with doctors from the British Cardiovascular Society, in which only 16% of them discussed sexual functioning with their patients^(18,34)^.

Associated with these data of low training of healthcare professionals for sexual counseling, many patients hospitalized for ACS experience a reduction in sexual desire and satisfaction due to fear of a new heart attack or sudden death, insufficient knowledge to resume sexual activity, lack of sexual counseling by healthcare professionals and anxiety^(30,35)^. These data are demonstrated in a systematic review that included eight studies that assessed women’s perception of sexual function after acute myocardial infarction (AMI)^(30)^ and also in a study with 128 patients with heart disease, in which anxiety played a mediating role in the relationship with physical health^(35)^.

In the study carried out in Turkey mentioned above, nurses listed some solutions for increasing the frequency of sexual counseling, such as ensuring privacy, choosing simple expressions using expressions such as “your partner”, in addition to using educational materials, such as CD, DVD, videos and specific guides for guidance in sexual counseling^(18)^. Within this perspective, in which the present study was carried out, a video was developed to facilitate sexual counseling by healthcare professionals with the main information regarding sexual activity after ACS. Studies show that patients who are counseled about sexual activity have better sexual resumption, desire and satisfaction^(18,32)^.

The video’s general topics were arranged in accordance with the non-pharmacological guidelines found during the narrative literature review. The first topic discussed in the video was the need to talk to a healthcare professional about the topic in question and reinforce the role of these professionals in clarifying any doubts that patients present, including about sexual activity, with the aim of reducing patients’ fear and trepidation in talking about the topic.

The second topic discussed was in relation to maintaining a healthy lifestyle, such as physical activity, healthy eating and medication adherence after hospitalization, essential subject in any guidance for secondary prevention of CVD and which is directly related to adequate sexual functioning, as a diet rich in calories and carbohydrates, lack of physical activity, tobacco and alcohol consumption are associated with worsening sexual quality^(36-38)^.

The third topic addressed and which is supported by national and international guidelines is the time for resuming sexual activity, with the following recommendations: gradual resumption after seven days of hospital discharge in patients without clinical signs of ventricular dysfunction; the importance of maintaining a calm environment and being rested for sexual activity; and initially opt for positions that require less energy expenditure and facilitate breathing as well as guidance regarding the clinical signs that should be noticed by patients during and after sexual activity^(33,37,39)^.

These guidelines are based on the American Heart Association’s sexual counseling guideline, which recommends sexual activity for those who can perform physical activities with expenditure of 3 to 5 MET (metabolic equivalent of task) and who do not present angina, excessive dyspnea, changes in electrocardiogram, cyanosis, hypotension, or arrhythmias^(12)^.

The last topic covered was in relation to medications that can alter sexual functioning and the use of medications that can aid in penile erection or substances that can be used to improve vaginal lubrication. Some medications used to treat CVD and the pathophysiology of coronary disease itself can compromise erectile function quality and vagina dryness, and patients are advised not to discontinue medications and only use medications that facilitate penile erection after appointment with healthcare professionals^(12,37,40)^.

After identifying the topics to be addressed in the video, it was developed and submitted to content evidence analysis by a heterogeneous group of experts with different knowledge and CVR was calculated, which supports recommendations in the literature, in which this score has been considered more accurate and rigorous than other calculations such as Content Validity Index^(41)^.

The last step of this study was video analysis by the target audience, which demonstrated excellent levels of agreement and positive opinions regarding the study topic and the way the video was demonstrated, and can be considered as a health education instrument for a safe sexual activity practice. The authors of this study chose to analyze the target audience’s opinions to increase the level of validity evidence even before analyzing the effects of this video on sexual satisfaction and feelings regarding sexual practice.

Analysis of content validity evidence by experts and the basis given by responses from the target population have been indicated by authors in the area of instrument or even technology development, as it allows the subjects covered in it to have a close relationship with the target population’s needs^(24,41)^. In the case of this study, assessment was carried out by patients admitted for coronary artery disease, since many of these patients experience feelings of fear and anguish when resuming sexual activity after AMI.

### Study limitations

The video developed reached an adequate level of content validity evidence both in the assessment of experts and in the analysis of lay people; however, the target population was not asked about their level of education, i.e., the level of educational training of this population was not assessed, only whether they knew how to read and write, which can be considered a limitation of this study.

### Contributions to nursing, health, or public policy

The educational video can be used as an educational tool by the multidisciplinary team for patients hospitalized for ACS as a way to equip these patients to resume sexual practice.

## CONCLUSIONS

Guidance topics for promoting safe sexual practice were identified through a narrative literature review, which allowed developing an educational video that was subjected to analysis of content validity evidence by a group of experts, obtaining a CVR higher than the critical value in all indicators assessed in the second round. The video validated by experts was subjected to analysis by the target audience and presented excellent CVR in all indicators, with positive opinions regarding the importance of the topic and the clarity of the video.

Based on the results of this study, this video can be used as a tool for health education for the patient population after hospitalization for ACS as well as being used in future research to assess the effects of this counseling on the return to sexual activity after hospitalization by ACS.
