# Supplementary material for: Sexual counseling for people with acute coronary syndrome: educational video development
Source: Rev Bras Enferm. 2024 Jul 29;77(3):e20230416. doi: 10.1590/0034-7167-2023-0416 (PMC11290733; doi:10.1590/0034-7167-2023-0416)
Supplement: Translation

# Translation: Aconselhamento sexual para pessoas com síndrome coronariana aguda: desenvolvimento de vídeo educacional

**DOI:** 10.1590/0034-7167-2023-0416pt

**Authors:** Laura Rizardi dos Santos Andrade, Lanay Dourado dos Anjos, Ana Paula Freitas Aguiar, Edvone Alves de Lima, Erika de Sá Vieira Abuchaim, Juliana de Lima Lopes, Camila Tákao Lopes, Vinicius Batista Santos

**Affiliations:** I Universidade Federal de São Paulo. São Paulo, São Paulo, Brasil; II Hospital Sírio-Libanês. São Paulo, São Paulo, Brasil; III Hospital Universitário São Paulo, Unidade de Cardiologia. São Paulo, São Paulo, Brasil; IV Hospital Israelita Albert Einstein. São Paulo, São Paulo, Brasil

**Keywords:** Educação Sexual, Sexualidade, Doenças Cardiovasculares, Educação em Saúde, Estudos de Validação

## Abstract

**Objetivo:**

avaliar as evidências de validade de um vídeo educativo sobre atividade sexual segura após síndrome coronariana aguda.

**Método:**

estudo em três fases: desenvolvimento de um vídeo; análise da validade de conteúdo por 11 especialistas; e análise da validade baseada nos processos de resposta por sete pessoas com doença coronariana. Calculou-se a razão de validade de conteúdo (CVR) com valores críticos para a segunda fase de 0,63 e para a terceira de 1,0.

**Resultados:**

o vídeo abordou a importância da retomada da atividade sexual e das posições que consomem menos energia, sinais clínicos de alerta, importância da adesão ao tratamento e do ambiente acolhedor para prática sexual. Obteve-se um CVR acima do valor crítico com um total de 4 minutos e 41 segundos.

**Conclusão:**

o vídeo educacional reúne adequadas evidências de validade de conteúdo, podendo ser utilizado como uma ferramenta para pacientes após síndrome coronariana aguda.

## INTRODUÇÃO

As doenças cardiovasculares (DCV) podem comprometer a vida da pessoa e de sua família tanto no aspecto fisiológico quanto no aspecto psicossocial. Pessoas que sofreram síndrome coronariana aguda (SCA) referem mudanças na qualidade de vida e aumento da dependência, além do isolamento social e evolução para estados de fragilidade com a progressão da doença. Uma das repercussões negativas das DCV e que impactam diretamente os aspectos psicossociais é a disfunção sexual^(1-3)^.

A resposta da função sexual foi inicialmente descrita por Willian Master e Virginia Johnson em quatro fases, sendo elas a excitação, o platô, o orgasmo e a resolução. Na década de 1970, Helen Kaplan entendeu que, antes da excitação, existe o desejo, sendo assim, eliminou a fase de platô, propondo o modelo trifásico da resposta sexual humana, contemplado pelas fases do desejo, da excitação e do orgasmo, podendo ser aplicado tanto em homens quanto em mulheres. Com base nos modelos de resposta sexual humana anteriormente descritos, a Classificação Internacional de doenças e o Manual Diagnóstico e Estatísticas dos Transtornos Mentais descreveu o ciclo de resposta da função sexual de forma linear em quatro fases: desejo; excitação; orgasmo; e resolução. Influenciado por fatores biológicos, psíquicos, sociais, históricos e culturais, esse modelo linear de quatro fases é mais característico da resposta sexual masculina^(4-5)^.

Em 2005, Basson descreveu um modelo circular de fases sobrepostas que caracteriza a resposta sexual feminina. Nesse modelo, partindo da neutralidade sexual, porém motivada e receptiva ao estímulo sexual, a mulher desencadeia uma excitação subjetiva. A vivência da excitação subjetiva leva ao desejo sexual e, com o aumento gradativo da intensidade da excitação e desejo, ocorre o prazer sexual, com ou sem orgasmo, levando à satisfação física e emocional^(5-6)^.

Diversos tipos de disfunções sexuais afetam a população geral em decorrência da falta de desejo sexual, excitação e/ou por dificuldades de atingir o orgasmo. Contudo, existem particularidades relacionadas às diferenças biológicas apresentadas pelos indivíduos, ou seja, pessoas com vagina ou com pênis podem apresentar maior prevalência de disfunção sexual em fases distintas da resposta sexual^(7)^.

A literatura demonstra na população em geral prevalência de 33,4% de problemas sexuais em pessoas com pênis^(8-9)^ e 45,7% em pessoas com vagina^(10)^. Nas pessoas com pênis, as disfunções sexuais que causam maior sofrimento são a disfunção erétil e a ejaculação precoce^(8-9,11)^. Nas pessoas com vagina, as disfunções sexuais mais comumente relatadas estão relacionadas ao desejo sexual hipoativo e disfunção orgástica, sendo a principal queixa relacionada à dor^(10-11)^. Entretanto, em pessoas com DCV, esses números tendem a subir, chegando a até 90% nas mulheres e 79% nos homens^(11)^.

Sabe-se que mulheres podem apresentar distúrbios sexuais diante da excitação, processo composto por uma série de mecanismos e características comportamentais, hormonais e morfológicas. Existem indícios de que as DCV e doenças metabólicas estão associadas com alterações na estrutura e função dos tecidos vaginais, impactando a *performance* sexual dessas mulheres^(11)^.

A disfunção erétil em homens apresenta forte relação como preditor de DCV e quadros subclínicos em homens assintomáticos. Assim, representa uma oportunidade em potencial para triagem da DCV nos homens com foco na detecção precoce e na prevenção de eventos deletérios, diminuindo, portanto, a morbidade e a mortalidade. Além disso, algumas medicações anti-hipertensivas usadas comumente em pessoas com DCV podem interferir negativamente no processo de ereção em si^(12-15)^.

Sendo a saúde sexual uma temática a ser considerada na população em geral e, em especial, naquelas com diagnóstico de alguma DCV, a educação em saúde exerce um papel fundamental para permitir uma orientação efetiva para que essa prática possa ser realizada de maneira satisfatória e com segurança. A educação em saúde é uma estratégia de promoção do cuidado ao paciente com o objetivo de atuar na prevenção e mitigar as complicações acarretadas por determinada doença, considerando o paciente como agente ativo e essencial para um tratamento eficaz, de forma a disponibilizar informações e orientações relevantes ao caso, visando promover a autonomia e o senso de responsabilidade sobre a própria condição clínica e as decisões a serem tomadas referentes ao tratamento^(16-17)^.

Estudos revelam que os profissionais de saúde demonstram dificuldade em abordar aspectos da sexualidade em pacientes com disfunção cardiovascular, especialmente entre aqueles pós-hospitalização por SCA^(18-20)^. A educação em saúde pode ser realizada em diversos cenários da prática dos profissionais, tais como visitas domiciliares, grupos, oficinas, vídeos e até mesmo com distribuição de materiais educacionais, como cartilhas educativas e até mesmo *blogs, sites* e redes sociais. Estudos sobre educação em saúde optaram pelo formato de vídeo por diversos motivos, tais como fácil distribuição e acesso, estímulos visuais que permitiram maior foco dos pacientes e obtiveram resultados positivos sobre o envolvimento dos mesmos dentro do regime de tratamento^(21-22)^.

Na área da cardiologia, uma pesquisa produziu um vídeo educacional voltado para pacientes que seriam submetidos ao cateterismo cardíaco. Entre os principais resultados, os pacientes apresentaram maior conhecimento sobre fatores relacionados ao procedimento e ao estado de saúde atual e obtiveram menores níveis de ansiedade, comparados aos pacientes do grupo controle. Tal modelo de intervenção apresentou diversas vantagens para a literacia em saúde dos pacientes, já que poderia ser visto na velocidade e quantidade de vezes desejada, facilitando a transmissão de conhecimento^(23)^.

Em levantamento feito pelos autores, não foram identificados estudos que desenvolveram e validaram um vídeo educativo para promoção da atividade sexual segura para pacientes que foram hospitalizados por SCA, e acredita-se que esse vídeo educativo possa ser implementado como uma ferramenta educacional para essa população.

## OBJETIVO

Desenvolver e avaliar as evidências de validade de um vídeo educativo sobre atividade sexual segura após SCA.

## MÉTODOS

### Aspectos éticos

Este projeto foi aprovado pelo Comitê de Ética e Pesquisa (CEP) com seres humanos da universidade, em conformidade com a Resolução do Conselho Nacional de Saúde/Ministério da Saúde n^o^. 466/12. Todos os participantes foram informados, por meio do Termo de Consentimento Livre e Esclarecido (TCLE), quanto ao objetivo da pesquisa e ao aspecto voluntário, bem como aos procedimentos da coleta de dados. Foram garantidos o anonimato e o sigilo aos participantes.

### Desenho e local do estudo

Trata-se de estudo metodológico de desenvolvimento e análise das evidências de validade de um vídeo educacional sobre a prática sexual segura após SCA. O estudo foi realizado em três fases: 1) desenvolvimento do vídeo; 2) análise das evidências de validade de conteúdo conforme a opinião de especialistas; e 3) análise das evidências de validade baseada nos processos de resposta por um grupo de pacientes hospitalizado por SCA em hospital universitário de grande porte na cidade de São Paulo contendo dez leitos de internação de cardiologia e seis leitos de terapia intensiva.

### Amostra e critérios de inclusão

Na segunda fase, participaram profissionais de saúde que realizaram pesquisas em relação à promoção da saúde sexual ou com experiência no processo de educação em saúde para os pacientes com DCV.

Na terceira fase, o vídeo previamente avaliado pelos especialistas foi apresentado e avaliado por pessoas leigas, alfabetizadas, maiores de 18 anos, hospitalizadas em hospital de grande porte na cidade de São Paulo por SCA e que não estavam hemodinamicamente instáveis, com recorrência de dor precordial ou em uso de fármacos vasoativos.

O tamanho amostral mínimo na segunda e terceira fase foi baseado nas recomendações de Lawshe para obtenção de uma razão de validade de conteúdo (CVR) crítica para adequada concordância entre os especialistas, sendo o mínimo de cinco especialistas^(24)^.

### Protocolo do estudo

Na busca da conceituação do problema, a primeira fase se baseou em revisão narrativa de literatura, que teve como objetivo mapear e identificar as principais orientações para a promoção da vida sexual de pessoas após SCA.

A busca e a seleção dos estudos foram norteadas pela técnica SPIDER: *Sample* (Amostra): pacientes adultos acima de 18 anos e com DCV; *Phenomenon of Interest* (Fenômeno de Interesse): orientações para a promoção da vida sexual; *Design* (Desenho do estudo): estudos de intervenção (ensaios clínicos, randomizado ou não randomizados, ensaios clínicos pragmáticos e estudos quase-experimentais); *Evaluation* (Avaliação): promoção da atividade sexual; *Research type* (Tipo de pesquisa): estudos observacionais, de revisões e *guidelines* ^(25)^.

Foram usados os descritores “*Sex Education*” e “*Cardiovascular Disease*”, em inglês, e “Educação Sexual” e “Doença Cardiovascular”, em português, nas fontes de dados da PubMed e Biblioteca Virtual de Saúde. Filtraram-se estudos publicados nos anos de 2016 a 2022.

Após a avaliação dos estudos recuperados por meio de leitura crítica dos títulos e resumos, eles foram lidos na íntegra por uma pesquisadora, sendo extraídos os seguintes dados: título; autores; ano de publicação; objetivo; tipo de estudo; e orientações não farmacológicas para a prática sexual segura.

Após a identificação das medidas não farmacológicas para a prática sexual segura, foi realizado um roteiro de vídeo por dois dos pesquisadores deste estudo com experiência na área de cardiologia e no estudo da sexualidade. O roteiro desenvolvido do tipo *storyboard* continha dois personagens fictícios, sendo um deles o paciente e o outro a enfermeira e, posteriormente, foi desenvolvido o vídeo educativo por meio do site *RenderForest^®^ Premium*. Foram abordadas no vídeo a importância da saúde sexual na qualidade de vida e na saúde e a importância da prática segura da atividade sexual, destacando-se as principais orientações não farmacológicas para manutenção de uma vida sexual saudável e funcional diante das limitações impostas pela doença.

Na segunda fase, o vídeo educativo foi submetido à avaliação de conteúdo pelos especialistas. Os potenciais participantes foram selecionados pela Plataforma Lattes e, com conhecimento dos próprios autores, foram enviados o convite para participação no estudo por e-mail e o TCLE por um *link* via *Google Forms* ^®^. Os especialistas avaliaram o vídeo no que diz respeito à clareza, relevância teórica e pertinência prática por meio de uma escala Likert (1 = discordo totalmente, 2 = concordo parcialmente e 3 = concordo totalmente).

A relevância teórica foi definida como “capacidade do item de ser consistente com o atributo definido e com as outras expressões que se relacionam ao mesmo atributo”, clareza, como “capacidade do item ser inteligível, com frases curtas, expressões simples e inequívocas”, e pertinência prática, como “elaboração de forma a avaliar o conceito de interesse em uma determinada população”^(26)^.

Na terceira fase, os potenciais participantes hospitalizados na unidade de internação de cardiologia foram abordados pela pesquisadora durante a hospitalização na unidade de cardiologia, tendo sido explicados os objetivos do estudo. Àqueles que aceitaram participar da pesquisa, foi aplicado o TCLE. Posteriormente, o vídeo foi apresentado aos pacientes por meio de um *tablet* e um fone *headset*, e foi solicitado que o avaliassem em relação à clareza das cenas e das informações, utilidade do vídeo, tempo de execução do vídeo e entendimento por meio de uma escala Likert (1 = discordo totalmente, 2 = concordo parcialmente e 3 = concordo totalmente). Para a complementação da avaliação, foi perguntado aos pacientes sua opinião sobre o vídeo e a temática abordada, tendo sido transcritas as respostas de forma literal.

### Análise dos resultados e estatística

Na segunda fase, para cada item do vídeo avaliado pelos especialistas, foi calculado o CVR da seguinte maneira:


$$CVR=\frac{ne-(N/2)}{N/2}$$

Assim, *ne* representa o número de especialistas que assinalaram a pontuação 3 e *N* relaciona-se ao número de especialistas^(24)^. Com base na devolutiva de 11 especialistas, o CVR crítico aceitável foi de 0,63 (p. 0,03)^(24)^.

Na terceira fase, para análise das evidências de validade baseadas nos processos de resposta, foi calculado o CVR, conforme descrito anteriormente, e, de acordo com a avaliação dos sete participantes, o CVR crítico foi de 1,0 (p. 0,008)^(24)^.

## RESULTADOS

Foram, inicialmente, identificados na revisão narrativa de literatura 385 artigos, sendo 62 desses avaliados para leitura completa dos artigos. Após a leitura crítica dos estudos, foram utilizadas quatro referências^(27-30)^ com as orientações para a retomada da atividade sexual segura após SCA, sendo que todas citaram o ambiente, a adesão medicamentosa e as posições que exigiram menor gasto de energia como orientações para a prática sexual.

As principais orientações oriundas dos artigos estão relacionadas ao ambiente seguro, à importância da adesão medicamentosa, da atividade física, da alimentação saudável e do reconhecimento dos sinais de alerta, a estar descansado para a prática sexual, a optar por posições que demandam menor gasto e facilitem a respiração, à retomada gradual da prática sexual, além das orientações quanto à necessidade da comunicação em relação a esta temática com os profissionais de saúde^(27-30)^. Demais informações dos artigos incluídos neste estudo estão dispostas no Quadro 1.

**Quadro 1:** Sumarização das orientações relacionadas à prática sexual. São Paulo, São Paulo, Brasil, 2022

Ref	Objetivo do estudo e tipo de estudo	Achados da pesquisa e tipos de aconselhamento
27	Fornecer informações essenciais para os profissionais de saúde iniciarem o aconselhamento sexual na prática e aplicável a homens e mulheres com DCV. Estudo de revisão	Medicação: estabelecer boa relação com paciente, orientar adesão medicamentosa, avaliar a raiz da disfunção e possibilidades de troca ou ajuste de dose; Ambiente: confortável e familiar para minimizar estresse cardíaco; Sinais de alerta: orientar relatar quaisquer sinais e sintomas; Treinamento físico: exercitar-se regularmente para diminuir o risco de eventos cardíacos; Intensidade e consumo de energia: dependente da capacidade física e estar bem descansado antes da atividade sexual; Posições: devem permitir conforto e facilitar a respiração.
28	Fornecer abordagens práticas baseadas em evidências para permitir que os profissionais de saúde discutam o aconselhamento sexual, ilustrado por vários cenários de caso. Estudo de revisão	Medicamentos: orientar relato de quaisquer efeitos colaterais e não cessar após surgimento de algum; Ambiente: confortável e familiar, descanso prévio a atividades sexuais, evitar refeições pesadas e consumo abusivo de álcool antes da relação sexual; Posição que propicie melhor conforto; Sinais de alerta: dor precordial, falta de ar extrema, frequência cardíaca elevada ou irregular, fadiga no dia seguinte à atividade sexual, tontura ou insônia; Atividade: atividade física regular conforme tolerância; Retomada da atividade sexual: iniciar com atividades que requerem menos intensidade/gasto energético, como abraços, beijos, carícias.
29	Descrever as últimas diretrizes publicadas pela *American Heart Association* sobre atividade sexual em pacientes com algum acometimento cardíaco, bem como sobre opções de tratamento de disfunção sexual. Estudo de revisão	Avaliação clínica (baixo risco de complicações cardíacas); Pacientes descompensados devem se abster de atividade sexual até estabilização; Caso seja notado algum tipo de disfunção sexual pelas medicações, não devem ser suspensas e as queixas devem ser avaliadas para descartar outras origens; Estar repousado antes da atividade sexual, evitar lugares e parceiros sexuais desconhecidos para reduzir estresse; Evitar alimentações pesadas e álcool; Preferência por posições que facilitem a inspiração.
30	Apresentar uma revisão de literatura abrangente que descreve os problemas sexuais de mulheres após infarto do miocárdio. Estudo de revisão	Déficit de conhecimento dos profissionais, baixa prevalência de orientações sobre a função sexual; Programas educacionais para terapia sexual: educação do paciente, reestruturação cognitiva, apoio emocional, orientação médica, aumentam frequência e qualidade de atividades sexuais após infarto do miocárdio; Fatores psicológicos (como ansiedade) agravados durante alterações fisiológicas durante sexo.

Com base no levantamento dos assuntos relacionados à orientação sexual após a SCA, foi realizado inicialmente um *storyboard* contendo dois personagens principais fictícios, sendo um deles o paciente após a alta hospitalar, denominado Pedro, e a enfermeira, Julia, que abordaram os tópicos citados no Quadro 1. Posteriormente, o vídeo foi elaborado no programa *RenderForest^®^ Premium,* contendo as falas, as imagens dos personagens em 3D e a narração, totalizando 34 telas, com 3 minutos e 48 segundos.

Após o desenvolvimento do vídeo, foi enviado o convite para 19 especialistas, com a devolutiva de 11 especialistas, sendo que 100% eram mulheres com idade média de 32 anos; 90,9% eram enfermeiras; 9,1% eram nutricionistas; 27% tinham pós-graduação *stricto sensu*; 72% tinham especialização; 90% tinham experiência na área de cardiologia; e 27% tinham experiência em cardiologia e sexualidade. Na avaliação geral do vídeo na primeira rodada, obteve-se um CVR de 0,4 em relação à quantidade de informações, 0,8, para o tempo de execução e utilidade, e 1,0, para pertinência prática das informações do vídeo. Na análise de cada cena, apenas duas telas não apresentaram um valor de CVR crítico quanto à clareza, três, em relação à relevância, e quatro, em relação à pertinência prática.

As principais sugestões na primeira rodada foram em relação a melhor contextualização da cena inicial, redação e ortografia, necessidade de apresentação da enfermeira no momento da consulta profissional, inclusão de tempo mínimo semanal para a realização da atividade física, acolhimento quanto à dúvida em relação à atividade sexual, melhor explicação das posições sexuais e esclarecimentos das medicações e risco de uso sem consentimento do médico e tomada de ação quanto ao aparecimento de sintomas isquêmicos durante e após a atividade sexual.

Após a modificação do vídeo, esse foi reenviado ao mesmo grupo de especialistas para nova avaliação, e foi obtido na avaliação geral em relação à quantidade de informações, ao tempo de execução e à utilidade um CVR 1,0. Na análise de cada cena, foi obtido um CVR superior a 0,80 em todas as cenas. O CVR médio atingido nessa rodada foi de 0,99 para clareza, 0,99 para relevância teórica e 0,98 para pertinência prática. O vídeo e o CVR final para cada cena em relação à clareza, relevância teórica e pertinência prática estão dispostos na Tabela 1.

**Tabela 1:** Versão final do vídeo e da razão de validade de conteúdo em relação aos indicadores avaliados. São Paulo, São Paulo, Brasil, 2023

Conteúdo	Cla	Rel	Pert
Vamos conversar um pouquinho sobre as preocupações de Pedro em relação à prática sexual após a alta do hospital	1,0	1,0	1,0
**Em uma reunião de amigos**	1,0	1,0	1,0
**Amigo:** Meu amigo Pedro, como você está se sentindo depois da alta do hospital?	1,0	1,0	1,0
**Pedro:** Estou bem, mas será que já posso voltar a ter relações sexuais?	1,0	1,0	1,0
**Amigo:** Acho que você deveria conversar com um profissional sobre isso na sua próxima consulta e esperar para voltar a ter relações sexuais.	1,0	1,0	1,0
**Pedro:** Acho que vou aguardar mesmo e perguntar na minha próxima consulta.	1,0	1,0	1,0
**No dia da consulta**	1,0	1,0	1,0
**Enfermeira:** Bom dia, Pedro! Sou a enfermeira Júlia. Que bom revê-lo. Como você está?	1,0	1,0	1,0
**Pedro:** Estou indo bem. Tomando todos os remédios que o médico me receitou.	1,0	1,0	1,0
**Pedro:** Comecei a caminhar 30 minutos pela manhã todos os dias. Estou me sentindo ótimo.	1,0	1,0	1,0
**Pedro:** E também estou comendo alimentos com pouco sal, evitando frituras e comendo mais frutas, verduras e legumes.	1,0	1,0	1,0
**Pedro:** Mas tem uma coisa que gostaria muito de perguntar e não sei como.	1,0	1,0	1,0
**Enfermeira:** Pedro, lembre-se que estou aqui para ajudar você a retomar sua vida com segurança após o infarto.	1,0	1,0	1,0
**Pedro:** Estou com medo de voltar a ter relação sexual. O que eu devo fazer?	1,0	1,0	1,0
**Enfermeira:** Olha, Pedro, esse é um assunto que podemos conversar com tranquilidade e clareza, sem julgamentos ou qualquer coisa do tipo.	1,0	1,0	0,8
**Enfermeira:** É recomendado que você espere pelo menos sete dias após a alta para a retomada das atividades sexuais.	1,0	1,0	1,0
**Enfermeira:** Importante que você comece devagar com toques, beijos e outras formas de prazer que você goste que gastem menos energia. Essas preliminares podem aumentar o desejo sexual tanto para você quanto para a pessoa com que você está.	1,0	1,0	1,0
**Enfermeira:** Uma alternativa para gastar menos energia é ficar em posição que você se movimente menos, ou seja, prefira ficar por baixo.	0,8	0,8	0,8
**Enfermeira:** Não importa qual o sexo. Veja nas fotos que a pessoa colorida é aquela que teve o infarto. Então, é recomendado que fique embaixo.	1,0	1,0	1,0
**Enfermeira:** Precisamos também saber reconhecer, em nosso próprio corpo, quais são os sinais de alerta.	1,0	1,0	1,0
**Enfermeira:** Fique atento se você sentir muito cansaço, dor no peito, e falta de ar durante ou após a atividade sexual.	1,0	1,0	1,0
**Enfermeira:** Caso sinta esses sintomas e eles melhorarem após a relação sexual, você deve procurar o médico ou o enfermeiro que está cuidando de você o mais breve possível.	1,0	1,0	1,0
**Enfermeira:** E tentar reduzir os esforços durante a relação sexual até conversar com esses profissionais.	1,0	1,0	1,0
**Enfermeira:** Mas caso não apresente melhora ao repouso, vá imediatamente ao pronto-socorro.	1,0	1,0	1,0
**Enfermeira:** Temos outras dicas que também são importantes. Anota aí, Sr. Pedro:	1,0	1,0	1,0
**Enfermeira:** Para melhorar sua relação sexual: escolha um ambiente tranquilo e calmo; procure estar previamente relaxado e descansado; tenha uma alimentação leve antes da relação sexual; e procure posições que você gaste menos energia.	1,0	1,0	1,0
**Enfermeira:** O médico irá receitar vários medicamentos importantes para você, e alguns podem ter alguns efeitos colaterais ruins, como	1,0	1,0	1,0
**Enfermeira:** Falta de vontade em ter relações sexuais, dificuldade em manter o pênis duro e pouca lubrificação na vagina.	1,0	1,0	1,0
**Enfermeira:** Mas não pare de seguir o tratamento, e caso haja algum problema, é só comunicar a enfermeira ou o médico que cuida de você.	1,0	1,0	1,0
**Enfermeira**: Para quem precisa, os lubrificantes podem ajudar na secura vaginal e também no prazer em si.	1,0	1,0	1,0
**Enfermeira:** Existem alguns remédios que podem ajudar o pênis ficar duro, mas esses medicamentos podem ser perigosos se usados sem autorização do médico.	1,0	1,0	1,0
**Pedro:** Ah, depois dessas informações, estou mais tranquilo, obrigado por tudo.	1,0	1,0	1,0
**Enfermeira**: Isso mesmo, Pedro! Você está indo muito bem e cuidando de si da melhor forma.	1,0	1,0	1,0
Para mais informações, acesse o site: www.educacor.unifesp.br	1,0	1,0	1,0

*Cla: clareza; Rel: relevância teórica; Per: pertinência prática.*

Após o vídeo ter sido analisado pelos especialistas e ter apresentado adequadas evidências de validade de conteúdo, o mesmo foi demonstrado para sete pessoas hospitalizadas por SCA com idade média de 61 anos, sendo 57% pacientes do sexo masculino. Entre os indicadores avaliados nessa fase, obtivemos um CVR igual a 1,0 para clareza das cenas, clareza das informações, utilidade do vídeo, tempo de execução e entendimento. Quando questionados quanto à opinião do vídeo e da temática da sexualidade, os pacientes relataram sentimentos positivos em relação ao vídeo e à abordagem realizada.

Na figura abaixo, encontram-se algumas telas do vídeo educativo desenvolvido para a orientação do paciente quanto à retomada da atividade sexual segura.

**Figura 1:** Exemplos na tela do vídeo educativo sobre atividade sexual segura após síndrome coronariana aguda

O vídeo educativo sobre atividade sexual segura após SCA pode ser assistido no QR *code* abaixo:

**Figura 2:** QR code do vídeo educativo sobre a atividade sexual segura após a síndrome coronariana aguda

## DISCUSSÃO

Segundo a Organização Mundial da Saúde, a sexualidade é uma das dimensões essenciais da vida humana e envolve múltiplos conceitos, como sexo, orientação sexual, identidade de gênero, erotismo, prazer, intimidade, reprodução e relações saudáveis. Sua complexidade e influência sobre a vida das pessoas são determinadas por fatores biológicos, psicológicos, sociais, econômicos, políticos, culturais e religiosos, além de experiências individuais e valores pessoais. A sexualidade é construída a partir do contexto em que as pessoas vivem e das interações que estabelecem com o ambiente em que se encontram^(31)^.

Sabendo da importância da dimensão sexual humana e seus impactos na qualidade de vida, é importante que profissionais estejam devidamente habilitados para realizar as orientações necessárias a cada dúvida de um paciente. É possível identificarmos estudos que demonstraram que os profissionais não possuem o hábito de abordar a sexualidade com os pacientes, seja por não se sentirem confortáveis e não considerarem a sexualidade como prioridade no cuidado ou por realmente não terem sido submetidos a um treinamento para abordar essa temática^(32-33)^.

Em estudo realizado na Turquia com 170 enfermeiros, foi identificado que os enfermeiros não abordavam a sexualidade nos pacientes com DCV por não considerarem a sexualidade uma prioridade no cuidado do paciente, por falta de privacidade na unidade de internação, por considerarem o assunto muito pessoal e por problemas culturais e religiosos^(18)^. Outros estudos também identificaram diversas dificuldades no aconselhamento sexual nessa população, como estudo na Jordânia, em que os enfermeiros se sentem com baixa confiança para a abordagem sexual, e *survey* realizado com médicos da sociedade de cardiologia da Inglaterra, em que apenas 16% deles discutem o funcionamento sexual com seus pacientes^(18,34)^.

Associado a esses dados de baixa capacitação dos profissionais de saúde para o aconselhamento sexual, muitos pacientes hospitalizados por SCA apresentam redução no desejo e na satisfação sexual por medo de um novo infarto ou morte súbita, conhecimento insuficiente para retomar a atividade sexual, falta de aconselhamento sexual pelos profissionais de saúde e ansiedade^(30,35)^. Esses dados são demonstrados em revisão sistemática que incluiu oito estudos que avaliaram a percepção das mulheres em relação à função sexual após o infarto agudo do miocárdio (IAM)^(30)^ e também em estudo com 128 pacientes com doença cardíaca, em que a ansiedade desempenhou um papel mediador na relação com a saúde física^(35)^.

No estudo realizado na Turquia citado anteriormente, as enfermeiras elencaram algumas soluções para o aumento na frequência no aconselhamento sexual, sendo elas garantir a privacidade, escolha de expressões simples com uso de expressões como “seu parceiro”, além do uso de materiais educacionais, como CD, DVD, vídeos e guias específicos para orientações no aconselhamento sexual^(18)^. Dentro desse prisma, no qual o presente estudo foi realizado, desenvolveu-se um vídeo para facilitar o aconselhamento sexual pelos profissionais de saúde com as principais informações em relação à atividade sexual após a SCA. Estudos demonstram que os pacientes que são aconselhados quanto à atividade sexual apresentam melhor retomada, desejo e satisfação sexual^(18,32)^.

Os tópicos gerais do vídeo se dispuseram de acordo com as orientações não farmacológicas encontradas durante a revisão narrativa de literatura. O primeiro assunto tratado no vídeo foi em relação à necessidade de conversar com um profissional de saúde sobre a temática em questão e reforçar a função desses profissionais no esclarecimento de qualquer dúvida que o paciente apresente, inclusive sobre a atividade sexual, tendo como objetivo a redução do medo e receio do paciente em conversar sobre a temática.

O segundo assunto abordado foi em relação à manutenção de um estilo de vida saudável, tais como atividade física, alimentação saudável e adesão medicamentosa após a hospitalização, assunto primordial em qualquer orientação para prevenção secundária de DCV e que apresenta relação direta com o adequado funcionamento sexual, pois a dieta rica em calorias e carboidrato, a falta de atividade física, o consumo de tabaco e álcool apresentam associação com a piora na qualidade sexual^(36-38)^.

A terceira temática abordada e que se sustenta nas diretrizes nacionais e internacionais é o tempo para a retomada da atividade sexual, sendo aconselhados: a retomada gradual após sete dias da alta hospitalar em pacientes sem sinais clínicos de disfunção ventricular; a importância de manter um ambiente tranquilo e estar descansado para a prática sexual; e optar inicialmente por posições que demandem menor gasto de energia e facilitem a respiração, além de orientações quanto aos sinais clínicos que devem ser percebidos pelo paciente durante e após a atividade sexual^(33,37,39)^.

Essas orientações estão pautadas no *guideline* sobre aconselhamento sexual da *American Heart Association*, que recomenda a atividade sexual para aqueles que podem realizar atividades físicas com gasto de 3 a 5 MET (taxa metabólica equivalente) e que não apresentam angina, dispneia excessiva, mudanças no eletrocardiograma, cianose, hipotensão, ou arritmias^(12)^.

A última temática abordada foi em relação aos medicamentos que podem alterar o funcionamento sexual e ao uso de medicamentos que podem auxiliar na ereção peniana ou substâncias que podem ser utilizadas para melhorar a lubrificação vaginal. Alguns medicamentos utilizados para o tratamento das DCV e a própria fisiopatologia da doença coronariana podem comprometer a qualidade da função erétil e a secura da vagina, sendo reforçada a orientação aos pacientes para não descontinuarem as medicações e somente utilizarem os medicamentos que facilitem a ereção peniana após a consulta com os profissionais de saúde^(12,37,40)^.

Após a identificação das temáticas a serem abordadas no vídeo, esse foi desenvolvido e submetido à análise das evidências de conteúdo por um grupo de especialistas heterogêneo com diferentes saberes, e foi calculado o CVR, o que corrobora as recomendações da literatura, em que esse escore tem sido considerado mais acurado e rigoroso do que outros cálculos, como o Índice de Validade de Conteúdo^(41)^.

O último passo deste estudo foi a análise do vídeo pelo público-alvo, o que demonstrou excelentes níveis de concordância e com opiniões positivas em relação à temática do estudo e à forma de demonstração do vídeo, podendo ser considerado um instrumento de educação em saúde para a prática segura da atividade sexual. Os autores deste estudo optaram em analisar as opiniões do público-alvo para aumentar o nível de evidências de validade antes mesmo de analisar os efeitos desse vídeo na satisfação sexual e sentimentos em relação à prática sexual.

A análise das evidências de validade de conteúdo por especialistas e o embasamento dado por respostas da população-alvo têm sido indicados por autores na área de desenvolvimento de instrumentos ou mesmo de tecnologias, pois permite que os assuntos abordados no mesmo tenham estreita relação com as necessidades da população-alvo^(24,41)^. No caso deste estudo, a avaliação foi realizada por pacientes admitidos por doença arterial coronariana, uma vez que muitos desses pacientes apresentam sentimentos de medo e angústia na retomada da prática sexual após o IAM.

### Limitações do estudo

O vídeo desenvolvido alcançou adequado nível de evidência de validade de conteúdo tanto na avaliação dos especialistas quanto na análise dos leigos, porém não foi interrogado à população-alvo sobre o grau de escolaridade, ou seja, não foi avaliado o nível de formação educacional dessa população, apenas se sabiam ler e escrever, o que pode ser considerado uma limitação deste estudo.

### Contribuições para as áreas da enfermagem, saúde, ou políticas públicas

O vídeo educacional pode ser utilizado como uma ferramenta educacional pela equipe multiprofissional para os pacientes hospitalizados por SCA como uma forma de instrumentalizar esses pacientes na retomada da prática sexual.

## CONCLUSÕES

Foram identificados os tópicos de orientação para a promoção segura da prática sexual por meio de revisão narrativa de literatura, o que permitiu o desenvolvimento de um vídeo educativo que foi submetido à análise das evidências de validade de conteúdo por um grupo de especialistas, obtendo um CVR superior ao valor crítico em todos os indicadores avaliados na segunda rodada. O vídeo validado pelos especialistas foi submetido à análise do público-alvo e apresentou excelente CVR em todos os indicadores, com opiniões positivas em relação à importância da temática e à clareza do vídeo.

Com base nos resultados deste estudo, este vídeo poderá ser utilizado como uma ferramenta para educação em saúde para a população de pacientes após hospitalização por SCA, bem como ser utilizado em pesquisas futuras para avaliação dos efeitos desse aconselhamento no retorno à prática sexual após a hospitalização por SCA.
